# Effects of low-level RF fields reveal complex pattern of magnetic input to the avian magnetic compass

**DOI:** 10.1038/s41598-023-46547-5

**Published:** 2023-11-15

**Authors:** Rachel Muheim, John B. Phillips

**Affiliations:** 1 Department of Biology, Lund University, Biology Building, 223 62 Lund, Sweden; 2 Department of Biological Sciences, Virginia Tech, Blacksburg, VA 24061-0406 USA

**Keywords:** Animal behaviour, Sensory processing, Animal migration, Biological physics

## Abstract

The avian magnetic compass can be disrupted by weak narrow-band and broadband radio-frequency (RF) fields in the lower MHz range. However, it is unclear whether disruption of the magnetic compass results from the elimination of the perception pattern produced by the magnetic field or from qualitative changes that make the pattern unrecognizable. We show that zebra finches trained in a 4-arm maze to orient relative to the magnetic field are disoriented when tested in the presence of low-level (~ 10 nT) Larmor-frequency RF fields. However, they are able to orient when tested in such RF fields if trained under this condition, indicating that the RF field alters, but does not eliminate, the magnetic input. Larmor-frequency RF fields of higher intensities, with or without harmonics, dramatically alter the magnetic compass response. In contrast, exposure to broadband RF fields in training, in testing, or in both training and testing eliminates magnetic compass information. These findings demonstrate that low-level RF fields at intensities found in many laboratory and field experiments may have very different effects on the perception of the magnetic field in birds, depending on the type and intensity of the RF field, and the birds’ familiarity with the RF-generated pattern.

## Introduction

A wide variety of animals use information from the Earth’s magnetic field for orientation and navigation^1–5^. Use of directional (‘compass’) information derived from the magnetic field was initially thought to be confined to migratory animals, but subsequent studies have shown that magnetic compass cues play an integral role in a wide variety of behaviours over multiple spatial scales^6–10^. The magnetic compass in many organisms, including birds, is light dependent^11–14^ and sensitive to the axial alignment (inclination) of the magnetic field^15^. It is suggested to be mediated by a photochemical reaction involving spin-correlated radical pairs in specialized photoreceptors, i.e., the radical-pair mechanism^16,17^. The Earth’s magnetic field is proposed to affect the interconversion between the singlet and triplet excited states of the radical pairs, which in turn modulates downstream signaling. Such photo-sensitive magnetoreceptors arranged in an ordered array, for example in the retina, will differ in response to light depending on their alignment relative to the magnetic field. As a consequence, in animals in which these specialized photoreceptors reside in the retina, the magnetic field may be perceived as a visual pattern superimposed on the animal’s surroundings or mediated by a separate sensory processing channel^17,18^. The resulting magnetic input, referred to here as the magnetic modulation pattern, would be an axially symmetric, 3-D pattern of light intensity, color, and/or enhanced contrast centered on the magnetic field lines^8,17,19^.

Cryptochromes have been suggested to be the most likely candidate magnetoreceptors for such a light-dependent magnetic compass^17,18^. They are the only animal photopigments known to form radical pairs upon light excitation with lifetimes long enough for a magnetic field effect to take place. In birds, several cryptochrome genes have been identified in the retinas of a variety of species^20–22^. Growing evidence suggests that Cry4 is the most likely candidate receptor for the light-dependent magnetic compass in birds^23–29^. Still, there is to date no conclusive experimental evidence that a specific cryptochrome or cryptochromes is involved in the primary magnetoreception process in birds or any other vertebrate.

The most convincing evidence for involvement of a radical-pair mechanism in the primary magnetoreception process so far has come from experiments testing the effects of low-intensity, radio-frequency electromagnetic fields (RF fields; ~ 0.1 to 100 MHz in the low nT range) on magnetic compass orientation of animals. According to quantum mechanical theory, such RF fields aligned non-parallel to the static magnetic field may alter the quantum spin state of the radical pair system and thereby influence the animals’ perception of the magnetic field^18,30–34^. Indeed, effects of RF fields on magnetic compass orientation have been demonstrated in several organisms, including amphipods^35^, cockroaches^36^, turtles^37^, murine rodents^38^, and birds. In birds, such effects have been found in several species of migratory songbirds^34,39–45^. Magnetic compass orientation has also been shown to be sensitive to RF fields in non-migratory zebra finches, *Taeniopygia guttata*^10,14,46^, and domestic chickens^47^. The presence of vertically aligned broadband RF fields as weak as 0.1 nT have been shown to effectively disrupt magnetic compass orientation^34,40,43^. RF fields at the local Larmor frequency have also been shown to lead to disorientation in birds, with sensitivity thresholds as low as 2–3 nT^34,39,44^. Despite the empirical evidence, however, there is no consensus on how such weak RF fields at the Larmor frequency can have a significant effect on a radical-pair-based magnetic compass^18,43,48–50^. It also remains unclear whether changes in magnetic compass orientation result from the elimination of the magnetic modulation pattern or from changes that make the pattern unrecognizable to the birds.

**Figure 1:**
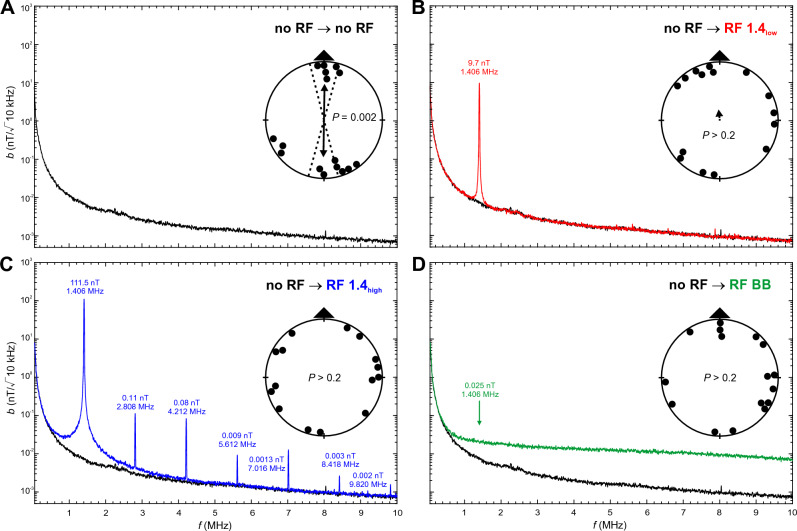
Magnetic compass orientation of zebra finches trained in the ambient RF environment (no RF). (**A**) Orientation of birds tested under the training condition (no RF; in black). (**B**) Orientation of birds tested in the presence of a low-intensity 1.4 MHz RF field (RF 1.4_low_; in red). (**C**) Orientation of birds tested in the presence of a high-intensity 1.4 MHz RF field (RF 1.4_high_; in blue). (**D**) Orientation of birds tested in the presence of a broadband RF field (RF BB; in green). Circular graphs show the orientation of the birds relative to the trained magnetic compass direction, indicated by the triangle on top of the circular graph. Each data point represents the orientation of an individual bird calculated as the vector sum of the time spent in each of the arms of the 4-arm maze during one of the 90 s probe trials. Arrows give the mean direction, with the length proportional to the mean vector length (radius of circle = 1). Double-headed arrows indicate bimodally distributed samples, two double-headed arrows indicate quadrimodally distributed samples. Significant distributions according to the Rayleigh test (P-values < 0.05) are shown with solid arrows and 95% confidence intervals (dotted lines). The mean vector of random distributions with 0.05 ≤ P ≤ 0.2 are shown with dashed arrows; no arrows are shown for totally random distributions with P > 0.2. See Table S2 for detailed statistics. Frequency spectra of the magnetic field intensities *b* of the RF fields are based on averages over 100 measurements taken at a frequency of 1 kHz, with a resolution bandwidth of 10 kHz. See Table 1 and Methods for more details on the frequency spectra.

**Figure 2:**
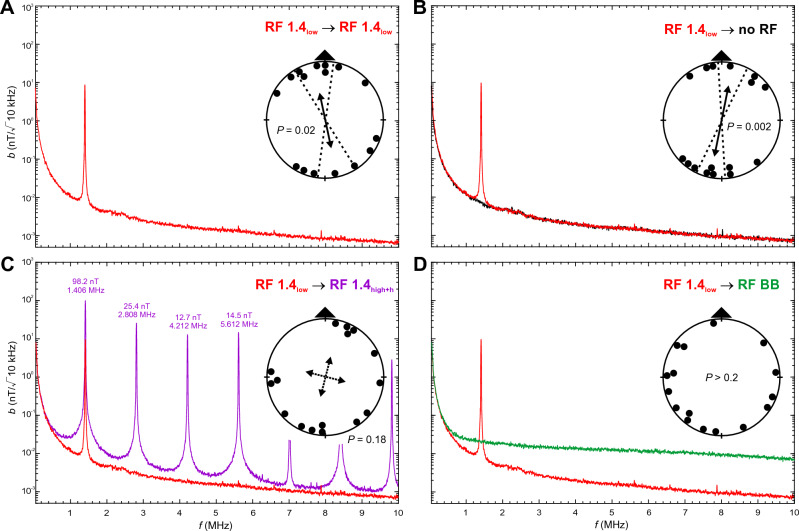
Magnetic compass orientation of zebra finches trained in the presence of a low-intensity 1.4 MHz RF field (RF 1.4_low_). (**A**) Orientation of birds tested under the training condition (RF 1.4_low_). (**B**) Orientation of birds tested in the ambient RF environment (no RF). (**C**) Orientation of birds tested in a high-intensity 1.4 MHz RF field with multiple harmonics (RF 1.4_high+h_; in purple). (**D**) Orientation of birds tested in the presence of a broadband RF field (RF BB). See Fig. 1 for a detailed explanation of graphs, and Table S3 for detailed statistics.

**Figure 3:**
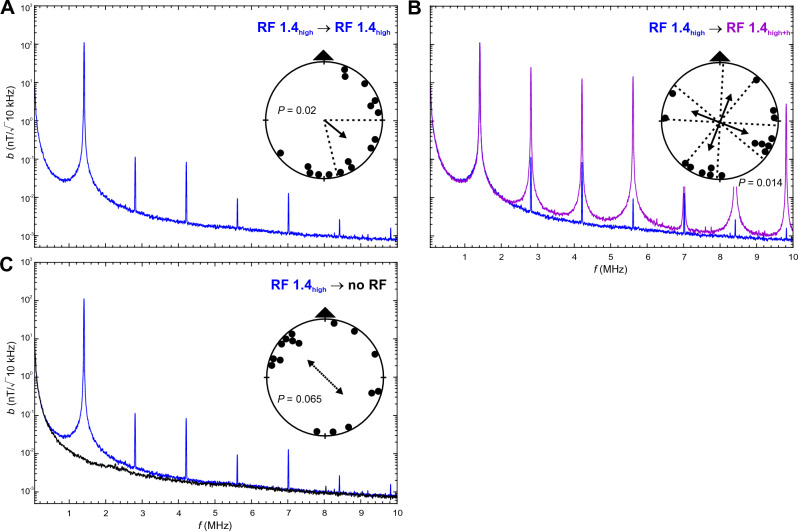
Magnetic compass orientation of zebra finches trained in the presence of a high-intensity 1.4 MHz RF field (RF 1.4_high_). (**A**) Orientation of birds tested under the training condition (RF 1.4_high_). (**B**) Orientation of birds tested in the presence of a high-intensity, multi-harmonic 1.4 MHz RF field (RF 1.4_high+h_). (**C**) Orientation of birds tested in the ambient RF environment (no RF). See Fig. 1 for a detailed explanation of graphs, and Table S4 for detailed statistics.

**Figure 4:**
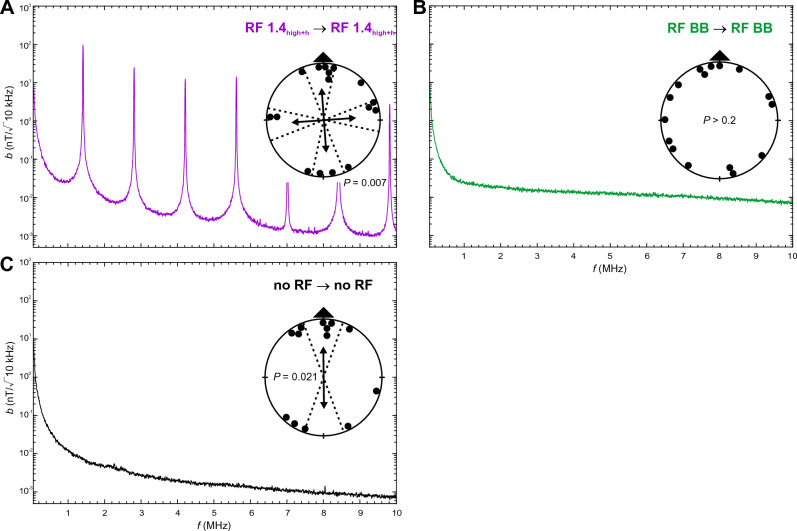
Magnetic compass orientation of zebra finches trained and tested in the presence of the same RF condition. (**A**) Orientation of birds trained and tested in the presence of a high-intensity, multi-harmonic 1.4 MHz RF field (RF 1.4_high+h_). (**B**) Orientation of birds trained and tested in the presence of a broadband RF field (RF BB). (**C**) Orientation of birds trained and tested in the ambient RF environment (no RF). See Fig. 1 for a detailed explanation of graphs, and Table S5 for detailed statistics.

Here we examined magnetic compass orientation of zebra finches trained and tested in spatial orientation experiments in a 4-arm maze in the presence of different RF fields. We showed previously that zebra finches can be trained to relocate a food reward in a cross maze using a light-dependent magnetic compass similar to migratory birds ^10,14^. As in migratory birds, magnetic compass orientation learned without RF present is abolished when zebra finches are tested under a Larmor-frequency RF field (1.406 MHz) at an intensity of ~ 260 nT ^10,14^; see also ^46^. In the present experiments, we trained and tested two sets of 16 adult male zebra finches under the following RF conditions (see Table 1 for wavelengths and frequencies of RF signals, Figs. 1, 2, 3 and 4 and Figs. S1 and S2 for frequency spectra, and “Methods” for calculation of intensity values):

- RF 1.4_low_: a low-intensity RF field with a peak frequency at 1.406 MHz, which is the Larmor frequency for the testing site; peak intensity *b* = 10 nT (/√10 kHz), total intensity *B*_*tot*_ = 15 nT above baseline;
- RF 1.4_high_: a high-intensity RF field with a peak frequency at 1.406 MHz with only minor harmonics; *b* = 111 nT, *B*_*tot*_ = 180 nT;
- RF 1.4_high+h_: a high-intensity RF field with a peak frequency at 1.406 MHz and multiple, prominent harmonics; *b* = 98 nT, *B*_*tot*_ = 260 nT;
- RF BB: a very low-intensity broadband RF field; *b* at 1.406 MHz = 0.025 nT, *B*_*tot*_ = 17 nT (0.05–25 MHz).

**Table 1:** Properties of the RF conditions measured in the center of the maze.

Peak frequencies and harmonics ≥ 1 nT		Magnetic field intensity at peak wavelengths	Total magnetic field intensity above baseline (no RF); 0.05–10 MHz	
	ƒ (MHz)	*b* (nT/√10 kHz)	*B*_*tot*_ (nT)	*B*_*rms*_ (nT)
No RF	–	0.007^a^	–	–
RF 1.4_low_	1.406	9.7	14.8	10.2
RF 1.4_high_	1.406	111.4	180.3	117.5
	2.808	0.11		
	4.212	0.08		
	5.612	0.009		
	7.016	0.013		
	8.418	0.003		
	9.820	0.002		
RF 1.4_high+h_	1.406	98.2	259.7	109.1
	2.808	25.4		
	4.212	12.7		
	5.612	14.5		
	7.016	1.1		
	8.418	5.4		
	9.820	2.8		
RF BB	–	0.025^a^	17.1^b^	1.6^b^

Given are peak frequencies, including harmonics ≥ 1 nT, and magnetic field intensity (magnetic flux density) at the primary frequency and harmonics. For ‘no RF’ and ‘RF BB’ the magnetic field intensity at 1.406 MHz is given. Total magnetic field intensity is the magnetic flux density above the baseline condition (‘no RF’) integrated over the frequency range of 0.05–10 MHz (0.05–25 MHz for RF BB), and given as the total magnetic field intensity, *B*_*tot*_, and root-mean-square magnetic field intensity,* B*_*rms*_. Note that we only refer to *B*_*tot*_ values in the text. See Figs. 1, 2, 3 and 4 and Figs. S1 and  S2 for frequency spectra of the RF fields for each condition, and the supplementary information for more details on the measurements and calculations of *B*_*tot*_ and *B*_*rms*_.

^a^Measured at 1.406 MHz.

^b^Frequency range 0.05–25 MHz.

As a control condition, we used the ambient RF environment in the absence of any introduced RF field (no RF). Two characteristics of the ‘no-RF’ condition may be important: (i) the intensity of RF was lower than in any of the experimental treatments, and (ii) any effect of the low levels of RF on the perception of the magnetic field would be familiar to the birds based on their previous experience.

For each experimental condition, we trained subgroups of four birds to find a food reward at mN, mE, mS, or mW. For each trained direction, one bird was subsequently tested with mN aligned towards gN, one bird with mN aligned to gE, one bird with mN aligned to gS, and one bird with mN aligned to gW to obtain all possible combinations of trained directions and test fields (see “Methods” and Table S1 for more details on experimental test procedure). The directional preference of each bird was calculated as the vector sum of the time spent in the four arms during the 90 s probe trial without food reward (see “Methods” for details on the data analysis). The use of this symmetrical testing format makes it possible to partition the variability in the distribution of bearings into an ‘absolute’ or ‘topographic’ component, a ‘spontaneous magnetic’ or ‘untrained magnetic’ component, and a ‘trained magnetic’ component^7,51^. In the absence of a response to the magnetic field, the resulting distributions from the group of birds tested under the same experimental condition were uniformly distributed (i.e., indistinguishable from random; see distributions of responses exhibited by birds exposed to broadband RF fields in training and/or testing). Consequently, the behavioral assay using the 4-arm maze made it possible to distinguish unimodal, bimodal, quadrimodal and ‘random’ distributions (see “Results and discussion” for discussion of the significance of these different types of responses).

## Results and discussion

### Zebra finches use a magnetic compass to relocate a food reward in a spatial orientation experiment

Zebra finches trained in the spatial orientation assay in the absence of any introduced RF field (no RF; Fig. 1, Table S2) and tested under the same condition were significantly oriented along the trained magnetic compass axis (no RF; Fig. 1A); the ‘topographic’ and ‘untrained magnetic’ components of the distribution of bearings were indistinguishable from random. The same was true for birds tested in a final control experiment (Fig. 4C; Table S5). As shown in earlier studies with zebra finches in the 4-arm maze^10,14^, the birds are able to learn the axis of a food reward (not present in the final probe trials) using directional information provided by the magnetic compass. They achieve this not only when trained to magnetic North or South, as shown previously^10,14^, but also when trained to magnetic East or West. These findings add to the credibility of this approach and further establish conditioned responses as an alternative to the traditional orientation experiments with migratory birds used to address similar questions.

### Larmor-frequency and broadband RF fields alter the perception of the magnetic field and lead to disorientation

Birds trained in the ambient RF environment (no RF) and tested in a low-intensity RF field at the Larmor frequency of 1.4 MHz (RF 1.4_low_: *b* = 10 nT, *B*_*tot*_ = 15 nT) were no longer oriented with respect to the trained magnetic compass axis (Fig. 1B). The zebra finches were also disoriented when tested under a high-intensity RF field at 1.4 MHz (RF 1.4_high_: *b* = 112 nT, *B*_*tot*_ = 180 nT; Fig. 1C) or a broadband RF field [RF BB:* b* = 0.025 nT at 1.406 MHz, *B*_*tot*_ = 17 nT (0.05 to 25 MHz); Fig. 1D]. In all three treatments in which birds were exposed to one of the RF stimuli (Fig. 1B–D), the distributions were uniformly distributed and there was no evidence of unimodal, bimodal, or quadrimodal clustering of bearings relative to the magnetic field (p > 0.20, Rayleigh test).

The disorientation of birds trained in the ambient RF environment and tested under one of the RF conditions shown in Fig. 1B–D suggests that both Larmor-frequency and broadband RF fields disrupt magnetic compass orientation by either eliminating or changing the magnetic modulation pattern, making it unrecognizable to the birds. This is in line with previous studies reporting disorientation in zebra finches, chickens and migratory songbirds tested in the presence of various RF fields^10,14,34,39–47^. The disorientation of the zebra finches tested under the extremely low Larmor-frequency RF field with peak intensity of only 10 nT (RF 1.4_low_) agrees with findings in garden warblers, *Sylvia borin*, in which the threshold for effects of RF fields at the Larmor frequency was found to be around 2–3 nT peak intensity^44^, and in European robins in which the threshold was between 5 and 15 nT^39^. Similarly, a broadband RF field with an intensity of as little as 0.025 nT at the Larmor frequency and a total intensity *B*_*tot*_ of only 17 nT above baseline was strong enough to disrupt the magnetic compass of the zebra finches. It confirms previous findings showing that broadband RF fields of very low intensities can disrupt magnetic compass orientation in birds^34,40,43^.

### Low-intensity Larmor-frequency RF field (RF 1.4_low_) degrades, but does not qualitatively alter, the perception of the magnetic field

To distinguish whether the disorientation of the birds trained under no RF and tested under RF 1.4_low_ resulted from elimination of the magnetic modulation pattern or from changes in the pattern that made it unrecognizable, we trained the birds in the presence of RF 1.4_low_ and subsequently tested them under the same condition. Interestingly, birds trained and tested under RF 1.4_low_ were significantly oriented along the trained magnetic compass axis (Fig. 2A, Table S3). Birds trained under RF 1.4_low_ were also well oriented when tested in the ambient RF field (no RF; Fig. 2B).

The ability of birds to orient under RF 1.4_low_ after training in the same RF field suggests that this condition does not eliminate directional information from the magnetic compass altogether. Rather, it appears to degrade the magnetic modulation pattern, without qualitatively altering the directional information obtained from the pattern, so that birds trained without exposure to this RF condition either had difficulty extracting directional information and/or did not recognize the magnetic modulation pattern as that associated with the food reward. Still, the resulting pattern is similar enough to the ‘no RF’ condition that birds trained in the low-intensity Larmor-frequency RF fields were able to orient along the trained magnetic axis in the ‘no RF’ condition, but not vice versa. It suggests that the birds exposed to RF 1.4_low_ in training were able to recognize the similar, but less degraded, pattern when tested in the ‘no RF’ condition. In part, this may reflect their familiarity with this pattern based on prior experience before the start of the present experiments.

Recognition of and/or familiarity with the magnetic modulation pattern may play an important role, at least when the pattern the birds are exposed to in testing is a less degraded version of the training pattern (i.e., RF 1.4_low_ → no RF). In a previous study, European robins pre-exposed to different RF fields, including a 1.315 MHz Larmor-frequency field at 15 nT, were not able to orient under the same RF condition immediately after being pre-exposed to the same RF field^42^. The 15 nT RF field used in Wiltschko et al.^42^ was slightly stronger than the 10 nT field that we used, thus the differences in responses could be due to a sensitivity threshold, alternatively species-specific differences in the sensitivity to Larmor-frequency RF fields, as indicated by Bojarinova et al.^45^. Considering that magnetoreception of the avian compass depends on the wavelength and intensity of light^11–13^, it is also possible that differences between the light conditions during pre-exposure and testing in Wiltschko et al.^42^ prevented the birds from becoming familiar with the changed magnetic modulation pattern. The robins were pre-exposed in holding cages illuminated by ‘white’ light, while the experiments were carried out under 565 nm green light in orientation funnels. If the resulting magnetic modulation patterns were different enough, familiarity with one of the patterns would not have enabled the birds to recognize the directional information available in the other pattern. We might expect that pre-exposure duration is also an important variable, if the ability to orient after pre-exposure to an RF field is based on a similar mechanism as the ability of migratory birds to orient in magnetic fields with intensities much weaker or stronger than the Earth magnetic field (~ 23–65 μT) after pre-exposure to those magnetic field intensities^52,53^. European robins were shown to require at least two 8-h pre-exposure periods to a 4 μT magnetic field, interspaced by one experiment, to be able to orient under this condition^52^. In contrast, robins in a different study were able to orient in a 92 μT magnetic field after only 1 h of pre-exposure^53^, which could indicate that the directional information contained in the modulation pattern was stronger than the one under 4 μT. The 7-h pre-exposure to the 15 nT RF-field used in Wiltschko et al.^42^ might simply not have been long enough to allow the birds to familiarize with the changed pattern. In nature, migratory birds may instead of, or in addition to, adaptation, calibrate an unfamiliar magnetic modulation pattern with respect to celestial cues (i.e., polarized light patterns present at sunrise and sunset^54,55^) to be able to use the magnetic pattern to orient in the seasonally appropriate migratory direction. In contrast, zebra finches in the present study were rewarded for learning the direction of a food reward with respect to the magnetic modulation pattern, which does not require lengthy pre-exposure or calibration with respect to an external/global reference system.

Birds trained under RF 1.4_low_ and tested in the presence of the high-intensity, multi-harmonic RF field (RF 1.4_high+h_: *b* = 98 nT, *B*_*tot*_ = 260 nT; Fig. 2C) were disoriented, but showed a weak tendency to orient quadrimodally along the cardinal magnetic compass directions (14°/104°/194°/284°, r = 0.327, P = 0.183). The zebra finches were completely disoriented when tested in the presence of the broadband RF field (RF BB; Fig. 2D). Given that birds trained under RF 1.4_low_ were able to orient in the trained direction when tested under the same RF field, the disorientation of birds tested under RF 1.4_high+h_ and RF BB is unlikely to result from the failure of birds to learn the task. Instead, RF 1.4_high+h_ and RF BB appear to have altered or eliminated the magnetic modulation pattern so that birds during testing either had problems extracting directional information and/or did not recognize the pattern as that associated with the food reward.

### High-intensity, Larmor-frequency RF fields (RF 1.4_high_) qualitatively alter the perception of the magnetic field

Zebra finches trained and tested under the high-intensity RF field at the Larmor frequency with only minor harmonics (RF 1.4_high_; Fig. 3, Table S4) were significantly oriented, but in a direction shifted by 128° relative to the magnetic compass direction in which they received the food reward during training (Fig. 3A). Birds trained under RF 1.4_high_ and tested in the presence of RF 1.4_high+h_ oriented quadrimodally with the two axes shifted clockwise by 21° from the cardinal magnetic compass directions (Fig. 3B; see also Fig. 4A below). Zebra finches trained under RF 1.4_high_ and tested in the natural RF environment (no RF; Fig. 3C) were disoriented, but showed a tendency to orient axially along the same shifted direction relative to the trained magnetic compass direction (134°/314°, r = 0.411, P = 0.065) as the other two groups trained under RF 1.4_high_. The three distributions did not differ from each other (Watson U^2^ tests: all P > 0.05).

The oriented response of birds exposed to RF 1.4_high_ (with harmonic intensities less than 1/1000^th^ of the intensity at the Larmor frequency) during both training and testing suggests that the magnetic modulation pattern may have been qualitatively altered, but still provided the birds with directional magnetic compass information relative to which they can orient. Since the distribution of bearings relative to mN (i.e., any untrained magnetic component of the responses) of the group of birds trained and tested under RF 1.4 _high_ is not significant (P = 0.577; Table S4), it is unlikely that the unimodal orientation is comparable to the ‘fixed’ magnetic responses reported in migratory birds^56^.

The inability of birds trained under RF 1.4_high_ to use the magnetic compass to orient in the trained direction when tested under ‘no RF’, as well as the inability of birds trained under ‘no RF’ to use the magnetic compass to orient under RF 1.4_high_, further suggests that, although the high-intensity Larmor-frequency RF field did not completely abolish the magnetic modulation pattern, this pattern differed enough from the pattern perceived in the natural (no RF) environment to substantially affect the birds’ perception of the magnetic field.

The unimodal orientation shifted 128° clockwise relative to the trained magnetic compass direction of birds trained and tested in the RF 1.4_high_ condition is intriguing. Although the explanation for this dramatic change in behavior is unclear, the unimodality in this condition suggests that exposure to RF 1.4_high_ produced a magnetic modulation pattern that is less ambiguous with respect to opposite directions along the same magnetic axis than the pattern available to the birds in the ‘no RF’ condition. However, this does not explain the shift in axis of orientation, which suggests that the altered pattern may include a reversed component relative to the pattern that the birds had associated with the magnetic direction of the food reward. Landler et al.^37^ found that exposure to a Larmor-frequency RF field at peak intensities between 30 and 52 nT during acclimation and testing caused a reversal in the direction of spontaneous magnetic alignment by yearling snapping turtles, *Chelydra serpentina*, relative to the spontaneous alignment of the turtles acclimated and tested without RF, which is consistent with the RF field altering, rather than eliminating, directional information obtained from the magnetic field. The reversal in the direction of alignment could have resulted from the difference in the magnetic modulation pattern the turtles experienced when exposed to the RF field relative to the pattern experienced prior to the experiments in the absence of RF exposure^37^, equivalent to the ‘no RF’ condition in the present experiments. However, it is unclear whether a similar effect could explain the ‘reversal’ in the direction of orientation in the present experiments since the birds trained and tested under RF 1.4_high_ were exposed to the same RF stimulus, and thereby to the same magnetic modulation pattern, during training and testing.

### Other frequency components added to the high-intensity Larmor-frequency RF fields (RF 1.4_high+h_) produces a strong quadrimodal component

Zebra finches trained and tested under the same high-intensity, multi-harmonic RF field (RF 1.4_high+h_: *b* = 98 nT, *B*_*tot*_ = 260 nT) exhibited quadrimodal orientation coinciding with the cardinal magnetic compass directions (Fig. 4A; Table S5), similar to birds trained under RF 1.4_low_ and tested under RF 1.4_high+h_ (Fig. 2C) and birds trained under RF 1.4_high_ and tested under RF 1.4_high+h_ (Fig. 3B).

The origin of the quadrimodal orientation is difficult to identify. Because the birds were tested with the magnetic field along the cardinal compass directions, we could not distinguish whether the birds were orienting with respect to magnetic or topographic cues (Tables S4 and S5), i.e., exhibiting a fixed magnetic response like the fixed alignment of migratory birds^56^ or a fixed response to the four arms of the maze. However, the uniform distributions of bearings in all three experimental conditions in which the birds were exposed to broadband RF fields, including experiments in which the birds were given the same broadband exposure in training and testing (Figs. 1D, 2D, 4C), suggests that quadrimodal orientation, when it did occur, i.e., when birds were exposed to the high-intensity, multi-harmonic 1.4 MHz RF field (RF 1.4_high+h_; Figs. 3B, 4A), may not have resulted from the elimination of the magnetic modulation pattern. If birds prevented from using magnetic cues relied on topographic cues associated with the 4-arm maze, we would expect similar responses from birds tested under RF 1.4_high+h_ and RF BB. It is possible, therefore, that the magnetic modulation pattern, rather than being totally devoid of any directional information, may have contained a strong component of radial symmetry in the presence of RF1.4_high+h_, preventing the birds from distinguishing among the cardinal compass directions. Further research will be needed to distinguish between these two possibilities, i.e. to determine if the quadrimodal orientation was a response to the altered magnetic modulation pattern and/or to the shape of the 4-arm maze.

### Extremely weak broadband RF noise totally abolishes directional information from the magnetic compass

Birds trained and tested under the same broadband RF field (RF BB: *b* = 0.025 nT at 1.406 MHz, *B*_*tot*_ = 17 nT [0.05 to 25 MHz]) were totally disoriented and their responses contained no sign of axial or quadrimodal symmetry relative to the trained magnetic compass direction (Fig. 4B; Table S5). This was also the case when birds trained in the absence of RF (no RF; Fig. 1D) or under RF 1.4_low_ (Fig. 2D) were tested under the broadband RF field, as described above.

The complete disorientation suggests that the broadband RF stimulus eliminated any discernable directionality from the magnetic modulation pattern and thereby completely eliminated directional information from the magnetic compass, even though the intensity was as little as 0.025 nT at the Larmor frequency and the total intensity *B*_*tot*_ was only 17 nT above baseline. This is in contrast to changes in the magnetic modulation pattern when birds were exposed to the Larmor-frequency RF fields which all retained some directional information from the magnetic compass (see above). These findings confirm the earlier work in migratory birds showing that exposure to low level broadband RF fields completely eliminated magnetic compass orientation^34,40,43^, while extending this work to show that, at least in zebra finches, the disorientation caused by broadband RF fields results from the complete elimination of the magnetic modulation pattern, rather than merely altering the pattern so that it was unfamiliar to the birds.

## Summary and conclusions

**Figure 5:**
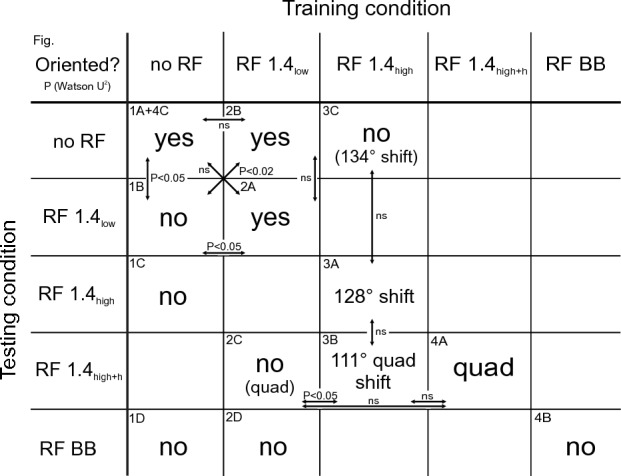
Summary of effects of different RF fields used in training and/or testing on magnetic compass orientation of zebra finches. ‘yes’: significant orientation (Rayleigh test: P < 0.05) towards trained magnetic compass direction/axis. ‘no’: no significant orientation (Rayleigh test: P ≥ 0.05). ‘shift’: significant orientation, but shifted by the given number in degrees relative to the trained magnetic compass direction. ‘quad’: quadrimodally oriented group of birds. Tendencies are given in brackets. P-values of the Watson U^2^ test are given in the comparisons between conditions. Figure numbers are given in the upper left corners.

Using a behavioural training assay with which we can not only study magnetic compass responses of birds tested in the presence of low-level RF fields, but also their ability to learn to orient in the presence of the same RF fields, we show that the perception of the magnetic field in birds is much more complex than previously thought. We provide clear evidence for distinct effects of exposure to different RF stimuli on the response of a radical-pair-based magnetic compass. Our results show that RF fields differing in intensity, frequency, and/or complexity have different effects on the perception of the magnetic field by zebra finches (see Fig. 5 for summary of results). The effects of exposure to the RF fields include degrading the magnetic modulation pattern without fundamentally altering the directional information, producing a discrete change that alters the directional information derived from the pattern, adding a quadrimodal component to the pattern, and eliminating the magnetic modulation pattern altogether.

Exposure to low-intensity Larmor-frequency RF fields (RF 1.4_low_; *b* = 10 nT, *B*_*tot*_ = 15 nT) during testing alters or degrades the magnetic modulation pattern, so that birds without previous exposure to the condition were disoriented (Fig. 1B). In contrast, birds trained under RF 1.4_low_, and thereby familiar with the condition, were able to orient relative to the magnetic field when tested under RF 1.4_low_ (Fig. 2A), indicating that the pattern retains directional information. The ability of birds trained under RF 1.4_low_ to orient along the trained magnetic axis when tested under no RF (Fig. 2B) suggests that exposure to RF 1.4_low_ degrades the magnetic modulation pattern, but does not fundamentally alter the directional information available from this pattern.

Exposure to high-intensity Larmor-frequency RF fields (RF 1.4_high_;* b* = 111 nT, *B*_*tot*_ = 180 nT) qualitatively alters the perception of the magnetic field, although it still provides the birds with directional information. Birds exposed to the RF 1.4_high_ fields in training and testing were able to orient with respect to the trained magnetic axis (Fig. 3A), but they appear unable to transfer magnetic compass information from the no RF to the RF 1.4_high_ condition (Fig. 3C), or vice versa (Fig. 1C). The inability to use the magnetic compass under RF 1.4_high_ suggests that these high-intensity Larmor-frequency RF fields qualitatively alter the magnetic modulation pattern so that it is no longer recognizable to birds that have not been exposed to the pattern previously. However, the oriented response of birds exposed to the same high-intensity RF stimulus (RF 1.4_high_) during both training and testing indicates that RF 1.4_high_ does not totally abolish the magnetic modulation pattern. Instead, the pattern still appears to contain directional information.

Exposure to high-intensity Larmor-frequency RF fields with multiple harmonics (RF 1.4_high+h_; *b* = 98 nT, *B*_*tot*_ = 260 nT) appears to add an orthogonal component to the magnetic modulation pattern that prevents birds from distinguishing between the trained and orthogonal-to-trained magnetic axes (Figs. 3B, 4A), resulting in quadrimodal magnetic orientation coinciding with the cardinal compass directions. Because cardinal compass directions and trained magnetic axes are confounded in these experiments, it is conceivable that the quadrimodal orientation is ‘fixed’ relative to the cardinal compass directions, rather than a response linked to the trained axis, and/or to the structure of the 4-arm maze.

Exposure to broadband RF fields (RF BB) at intensities of as little as 0.025 nT at the Larmor frequency (*B*_*tot*_ = 17 nT) prevented the birds from obtaining directional information from the magnetic field (Figs. 1D, 2D; see also^34,40,43^). Birds that were trained in the presence of such weak broadband RF fields were unable to derive directional information from the magnetic field, even when exposed to the same weak broadband RF field in training (Fig. 4B), which strongly suggests that RF BB completely eliminated the magnetic modulation pattern.

Taken as a whole, our findings provide compelling support for the radical-pair mechanism or similar quantum process, and expand our understanding in how birds perceive magnetic compass information in the presence of extremely low-level RF fields. More generally, these findings help to explain the difficulty in a variety of organisms of reliably eliciting magnetic compass responses in laboratory settings in the absence of electromagnetic shielding to screen out low-level RF fields^7,40,57,57,58^. Not only may the presence of RF fields from a variety of sources in the laboratory (e.g., computers, laboratory equipment, thermostats, ventilation motors, broadcast antennas; pers. obs.) affect experimental subjects’ perception of the magnetic field, but differences in the types and intensities of ambient RF fields both within and between laboratories may be important sources of uncontrolled variability in responses to magnetic cues. Moreover, the findings reported here suggest that further research is needed on the effects of electromagnetic fields in the vicinity of human habitation and other anthropogenic sources (power lines, radio antennas, etc.) on natural behavior of animals in the wild^59^.

## Methods

### Experimental animals

We trained and tested a total of 32 adult (> 6 months of age) male zebra finches, *Taeniopygia guttata*, from our own breeding stock. This study was carried out with the approval of Malmö-Lund Ethical Committee (permit nr. M 24-16) and conducted in accordance with Swedish legislation and the ARRIVE guidelines.

### Bird housing and experimental setup

All experiments were carried out between May and August 2016, and during May and November/December 2017, at Stensoffa Field Station, located 20 km from Lund, Sweden, in a remote area with very low levels of anthropogenic RF (Fig. S1). Throughout the experiment, birds were housed in a wooden building under full-spectrum light composed of natural light from two windows covered with a translucent film to prevent insight, and the addition of a full-spectrum lamp. The visually symmetric 4-arm (‘plus’) maze was centered in a pair of orthogonally aligned magnetic coils which produced an artificial magnetic field closely resembling the ambient magnetic field (inclination 69.8°, total intensity 50, 500 nT). The magnetic field could be directed towards any of the four maze arms (mN at gN, gS, gE or gW). The maze was illuminated by 522 nm green light (27 mW/m^2^, measured with a radiometer, model IL 1400 with detector SHD033; International Light Technologies, MA, USA), produced by an array of LEDs (OF-BLR5060RGB300, OPTOFLASH, Łódź, Poland). See Muheim et al.^10^ for a detailed description of the experimental setup.

### RF conditions

The vertically aligned experimental RF fields (see Table 1 for wavelengths and frequencies of RF signals, and Figs. 1, 2, 3 and 4 and Fig. S2 for frequency spectra) were produced by a loop antenna (ø 1.2 m) constructed from a coaxial cable with 2 cm of the shielding removed opposite the feed. It was attached horizontally underneath the testing table and powered by a function/arbitrary waveform generator (Agilent 33210A, 10 MHz, Santa Clara, CA, USA). The fields were regularly checked with an EMC EMI magnetic field probe (Probe 901 from E & H Near Field set #7405, EST Lindgren, St. Louis, MR, USA) connected to a spectrum analyzer (Agilent N9340B, Santa Clara, CA, USA). To produce the high-intensity RF fields (RF 1.4_high_, RF 1.4_high+h_) the signal was amplified with a broadband amplifier (Toellner TOE 7607, DC to 5 MHz, Toellner Electronic Instruments GmbH, Herdecke, Germany). In addition to amplifying the primary RF signal it also produced a number of harmonics (cf. Table 1). To determine if the harmonics played any role in the zebra finches’ responses, we compared effects of the high-intensity RF signal with (RF 1.4_high+h_) and without (RF 1.4_high_) the harmonics present. Reduction in the number and amplitudes of harmonics in the RF 1.4_high_ condition was produced by the addition of two low-pass RF filters (BLP-1.9+, Mini-Circuits, Brooklyn, NY, USA).

### Measurements of magnetic properties of RF conditions

Magnetic flux density $b(f,{\Delta f}_{0})$ for each RF condition was measured in the center of the maze. With the exception of the broadband RF field (RF BB; see below), all measurements were taken over the frequency range *ƒ* = 0.5–10 MHz *(∆ƒ* = 9950 kHz) at a frequency resolution of 1 kHz and a resolution bandwidth *∆ƒ*_0_ = 10 kHz. Measurements were taken in sample detection mode and each data point (*N* = 9951) was averaged over 100 measurements using trace averaging, i.e., the averaging of each trace with the previously swept data average for the same trace point (Spectrum Analysis Basics, Application Note 150, Agilent Technologies).

Total magnetic field intensity *B*_*tot*_ and root-mean-square magnetic field intensity *B*_*rms*_ for each RF condition were calculated by first subtracting the magnetic flux densities of the RF condition *b*_*rf*_ from the corresponding values of the baseline measurement *b*_*no_rf*_, resulting in the magnetic flux density above baseline $b\left(f,{\Delta f}_{0}\right)={b}_{rf}\left(f,{\Delta f}_{0}\right)-{b}_{no\_rf}\left(f,{\Delta f}_{0}\right)$ at each of the measured frequencies *ƒ*_*i*_. We then calculated the total magnetic field intensity *B*_*tot*_ and the root-mean-square magnetic field intensity *B*_*rms*_ over the frequency range of 0.05–10 MHz, using the following formulas:

$${B}_{tot}= \frac{1}{N}\frac{\Delta f}{{\Delta f}_{0}}\sum {b}_{i}({f}_{i},{\Delta f}_{0})$$

$${B}_{rms}=\sqrt{\Delta f}\sqrt{\frac{1}{N}} \sum {{(b}_{i}/\sqrt{{\Delta f}_{0}})}^{2}$$

Since the broadband RF field (RF BB) produced by our signal generator did not exceed frequencies above ~ 22 MHz (Fig. S2), we calculated the total and root-mean-square intensities of RF BB over the frequency range of 0.05 to 25 MHz. *B*_*tot*_ and *B*_*rms*_ therefore give the total magnetic field intensity for the broadband RF field.

### Training and testing procedure

In the late afternoons, individual birds were trained to relocate a hidden food reward at the end of one of the maze arms. The food reward was located in one of the four magnetic field directions (mN, mE, mS, or mW) under different experimental conditions (see Table 1). The birds were taken individually from their home cage and brought to the testing building where they were released in the center of the maze and allowed to explore the arena. Each time a bird hopped onto a wrong, empty tray it was punished by 5–10 s of darkness, before allowed to proceed. Once the bird found the food reward it was allowed to eat for 15–30 s, before being removed from the maze and returned to the home cage with access to full-spectrum light. The training trial was considered successful when a bird was able to find the food reward by entering no more than 8–10 arms. Birds that did not pass this threshold were excluded from further training the same afternoon. Birds that successfully found the reward were trained once again 30–90 min after the first training. Between the two training sessions, the location of the rewarded arm was rotated either clockwise or counter clockwise by 90° together with the alignment of the magnetic field. To qualify for a probe trial the following day, the total time it took an individual bird to successfully find the food reward in the two trainings had to be no longer than 8 min. Birds that found the reward in the first arm they visited in both of the trainings did not qualify for a probe trial, since pre-trials showed that they likely found the reward by chance and did not properly learn the task.

Birds that successfully passed the two training trials were tested in a probe trial the day after training. Testing procedures were identical to the training procedures, with the exception that there was no food reward in any of the trays. The birds were allowed to search the maze for 90 s (starting when the bird entered the first arm) without any interference by the tester. Calculation of a bird’s directional preference in each testing trial is described below.

### Experimental design

We trained and tested two sets of 16 zebra finches (32 birds in total) under the experimental conditions described in the main text (Table 1). For each experimental condition, groups of four birds were trained to find the food reward at mN, mE, mS, or mW. For each trained direction, one bird was subsequently tested with mN aligned towards gN, one bird with mN aligned to gE, one bird with mN aligned to gS, and one bird with mN aligned to gW to obtain all possible combinations of trained directions and test fields (see Fig. S3 for an illustration of the training and testing scheme). Within each of the two experimental sets of 16 birds, an individual bird was trained relative to the same magnetic compass direction, but tested in different magnetic field alignments. Within each test series, individual birds were repeatedly trained under a training condition and tested in probe trials under the different test conditions until each bird had one valid probe trial under each condition. Thus, each individual bird is only represented once in each experimental group.

The experimental testing scheme was as follows (see Table S1):

In set I, 16 birds were repeatedly trained in the natural RF environment without any artificial RF field (no RF). All birds were first tested under the trained condition without RF (no RF; test series 1a). Then, they were tested in pseudorandom order under RF 1.4_low_, RF 1.4_high_, and RF BB (test series 1b).

In set II, we trained and tested 16 other individuals in four consecutive series of experiments. In the first series (test series 1), the birds were trained in the presence of a low-intensity, 1.4 MHz RF field (RF 1.4_low_) and tested under four experimental conditions. In the first subset (test series 1a), about half of the individual birds were first tested under RF 1.4_low_ and then under RF 1.4_high+h_ and the other half was first tested under RF 1.4_high+h_ and then under RF 1.4_low_. In the second subset (test series 1b), about half of the birds of each group in test series 1a was first tested under RF BB and then under no RF and the other half was first tested under no RF and then under RF BB. In the second series of experiments (test series 2), the same individuals used in series 1 were either first trained and tested in the presence of RF BB and then RF 1.4_high+h_, or vice versa. In the third test series (test series 3), the birds were trained in the presence of RF 1.4_high_, and tested under RF 1.4_high_, RF 1.4_high+h_ and no RF in pseudorandom order. Finally, to test whether the birds were able to orient in the absence of any artificial RF fields, the individuals used in test series 1–4 were trained and tested in the natural RF environment (no RF; test series 4).

Within each of the test series, individual birds were repeatedly trained under a training condition and tested in probe trials under the different test conditions until each bird had one valid probe trial under each condition. Thus, each individual bird is only represented once in each experimental group.

### Data analysis and statistics

A bird’s movements during a probe trial was tracked with a custom video tracking program that automatically counted the number of frames that the bird spent in each of the four arms. The vast majority (> 95%) of birds tested in the maze were continuously active during the 90 s probe trial and repeatedly visited the four maze arms. It occurs almost never that a bird only visits one arm and then sits still there. Also, the birds almost always walk all the way to the end of the arm, hop onto the empty food tray and then walk out of the arm again, usually at an even pace. The time it takes a bird to walk all the way to the end of an arm and back out again is twice the time it takes a bird to walk only halfway into an arm and back out, so visits to the end of an arm do weigh twice a visit only halfway into an arm.

The orientation of an individual bird was calculated from the time (number of frames) it spent in each of the four maze arms during the 90 s trial (cf.^10,51^). The topographic directions of the four maze arms, i.e. 0°, 90°, 180°, and 270°, weighed by the number of frames the bird spent in each of the arms, were added using vector addition, which resulted in a topographic mean orientation for the individual bird^60^. A mean orientation can be calculated from grouped data with any number of groups, provided the mean vector length is adjusted for the grouping (cf.^60–62^). In the case of four groups, the mean vector length has to be multiplied with 1.1107. However, since the time spent in the different arms is not an independent measure, we did not use the individual mean vector length nor did we calculate any test statistics, but we simply calculated the mean direction by vector addition.

The resulting individual mean directions relative to topographic North were then recalculated relative to magnetic North (mN = 0°, taking into consideration that different individuals were tested in each of the four magnetic field alignments), and relative to the trained magnetic compass direction (correcting for whether the bird was trained to mN, mE, mS, or mW).

For each experimental condition, the mean orientation of the group of birds was calculated using vector addition from the individual mean directions, disregarding the individual mean vector lengths. For all groups, we determined whether an unimodal, bimodal or quadrimodal axial distribution best fitted the orientation data by calculating the mean vector length for the three distributions. To calculate the bimodal distributions, we doubled the individual mean angles, and to calculate the quadrimodal distributions, we quadrupled the individual mean angles^60^. For the distribution that best described the data, i.e. the distribution with the largest mean vector length, the Rayleigh test was performed to test for significance^60^. We used the 95% confidence interval to determine whether the distribution of directional responses from each group of birds was oriented relative to the trained magnetic direction, i.e., whether the trained direction was included in the 95% confidence interval of the distributions of birds in significantly oriented groups^60^. Watson U^2^-tests were used to test for differences between experimental groups^60^.

### Supplementary Information

Supplementary Information 1.

Supplementary Information 2.

## Data Availability

All data generated or analysed during this study are included in this published article and its supplementary information files.
