# Supplementary material for: Effects of low-level RF fields reveal complex pattern of magnetic input to the avian magnetic compass
Source: Sci Rep. 2023 Nov 15;13:19970. doi: 10.1038/s41598-023-46547-5 (PMC10651899; doi:10.1038/s41598-023-46547-5)
Supplement: Supplementary file 2 — Supplementary Information 2. [file 41598_2023_46547_MOESM2_ESM.pdf]

Supplementary Information

Effects of low-level RF fields reveal complex pattern of magnetic input to the avian magnetic compass

Rachel Muheim[1*] & John B. Phillips[2]

[1]Department of Biology, Lund University, Biology Building, 223 62 Lund, Sweden

[2]Department of Biological Sciences, Virginia Tech, Blacksburg, VA 24061-0406, USA

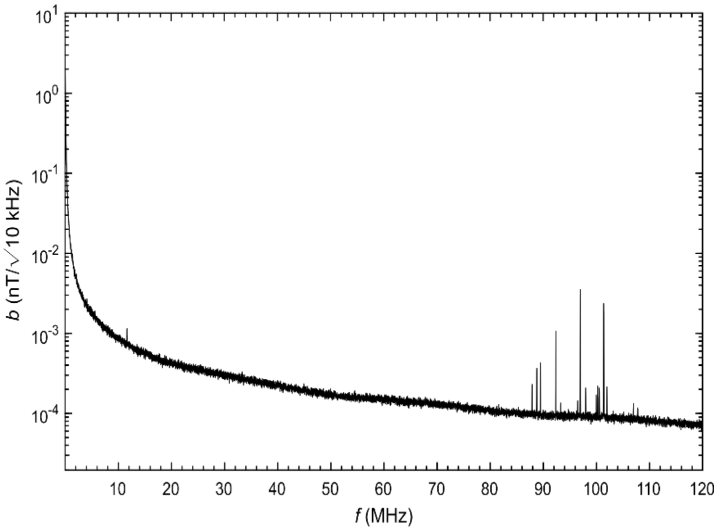

Supplementary Figure S1. Properties of ambient RF environment. Frequency spectrum of the magnetic field intensity *b* of the ambient RF environment at Stensoffa Field Station. Repeated measurements of the ambient RF environment at the station show that there is very little, if any, artificial RF noise below 85 MHz. The spikes visible between 85 and 110 MHz are FM radio stations (all <0.1 nT/√10 kHz). The RF field was measured between 50 kHz and 120 MHz at 10 kHz resolution (N=11995), each data point averaged over 50 measurements at a resolution frequency of 10 kHz. See Methods for details on calculations.

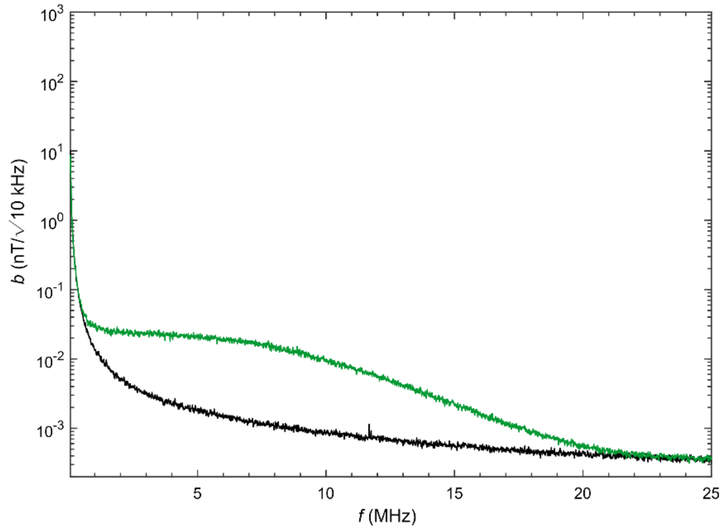

Supplementary Figure S2. Properties of broadband RF field (RF BB). Frequency spectrum of the magnetic field intensity of the broadband RF field (RF BB; in green) from 0.05 to 25 MHz, in comparison with the ambient RF field (no RF; in black). The RF fields were measured over the frequency range f = 0.5–25 MHz (Δf = 24,950 kHz) at a frequency resolution of 10 kHz and a resolution bandwidth Δf_0 = 10 kHz, with each data point (N = 2496) averaged over 50 measurements. See Methods and Table 1 for details.

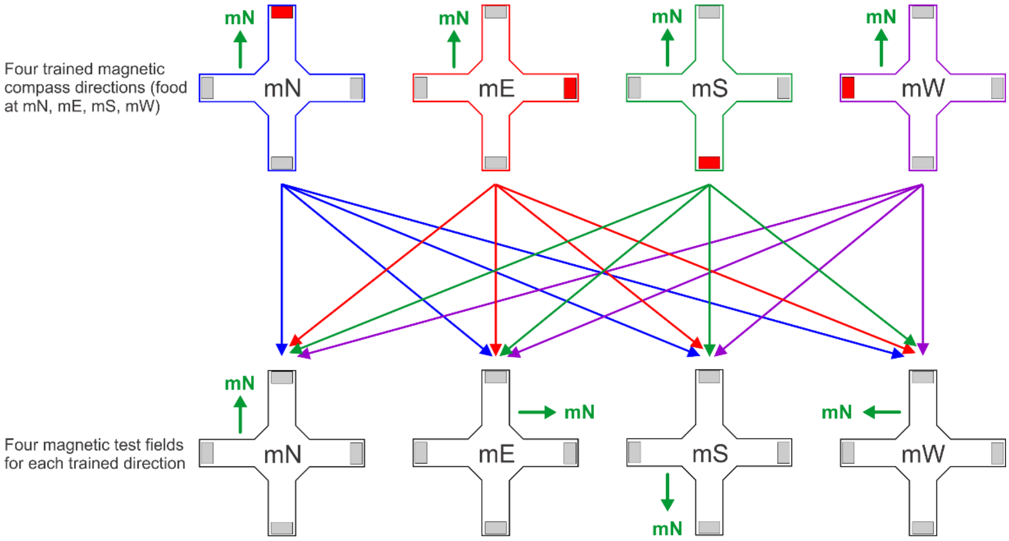

Supplementary Figure S3. Illustration of the training and testing scheme. For each experimental condition, groups of four birds were trained to each of the four trained magnetic compass directions [food reward (red box at end of arm) at mN, mE, mS, or mW; top row]. For each of the four trained directions, one bird was then tested in one of the four test fields (mN aligned towards gN, gE, gS, or gW; bottom row) to obtain all possible combinations of trained directions and test fields.

Supplementary Table S1. Experimental conditions used in the different test series during training and testing.

	Training conditions	Test conditions (number of birds first tested under test condition)		
Set I (n=16 birds)				
Test series 1a	No RF	No RF (n=16)		
Test series 1b	No RF	RF 1.4_{low} (n=6)	RF 1.4_{high} (n=5)	RF BB (n=5)
Set II (n=16 birds)				
Test series 1a	RF 1.4_{low}	RF 1.4_{low} (n=9)	RF 1.4_{high+h} (n=7)	
Test series 1b	RF 1.4_{low}	RF BB (n=8)	no RF (n=7)	
Test series 2a	RF BB	RF BB (n=8)		
Test series 2b	RF 1.4_{high+h}	RF 1.4_{high+h} (n=8)		
Test series 3	RF 1.4_{high}	RF 1.4_{high} (n=5)	RF 1.4_{high+h} (n=6)	no RF (n=5)
Test series 4	No RF	No RF (n=13)		

See Methods for explanations and Supplementary data for summary and more details.

Supplementary Table S2. Magnetic orientation of zebra finches trained in the ambient RF environment (no RF; Set I, test series 1; Fig. 1).

	Birds tested for magnetic compass orientation under the training condition (no RF; Fig. 1A)			Birds tested for magnetic compass orientation in the presence of a low-intensity 1.4 MHz RF field (RF 1.4_{low}; Fig. 1B)			Birds tested for magnetic compass orientation in the presence of a single-peak 1.4 MHz RF field (RF 1.4_{high}; Fig. 1C)			Birds tested for magnetic compass orientation in the presence of a broadband RF field (RF BB; Fig. 1D)		
Orientation	gN	mN	Trained MC direction	gN	mN	Trained MC direction	gN	mN	Trained MC direction	gN	mN	Trained MC direction
N	16	16	16	15	15	15	15	15	15	16	16	16
Mean direction	91°	96°/276°	1°/181°	18°	265°/85°	355°	110°	67°/247°	80°/260°	10°	52°/232°	78°
Mean vector length	0.261	0.352	0.601	0.382	0.403	0.188	0.250	0.358	0.287	0.340	0.114	0.310
Rayleigh test (P)	0.343	0.138	0.002	0.111	0.085	0.598	0.398	0.146	0.296	0.157	0.817	0.218

Orientation statistics for each experimental group are given relative to geographic North (gN), relative to magnetic North (mN = 0°) and relative to the trained magnetic compass (MC) direction. Sample size (N), mean direction, mean vector length as measure of scatter and P-value for the Rayleigh test is given for each experimental group.

Supplementary Table S3. Magnetic compass orientation of zebra finches trained in the presence of a low-intensity 1.4 MHz RF field (RF 1.4_{low}; Set II, test series 1; Fig. 2)

	Birds tested for magnetic compass orientation under the training condition (RF 1.4_{low}; Fig. 2A)			Birds tested for magnetic compass orientation in the ambient RF field (no RF; Fig. 2B)			Birds tested for magnetic compass orientation in the presence of a multi-harmonic 1.4 MHz RF field (RF 1.4_{high+h}; Fig. 2C)			Birds tested for magnetic compass orientation in the presence of a broadband RF field (RF BB; Fig. 2D)		
Orientation	gN	mN	Trained MC direction	gN	mN	Trained MC direction	gN	mN	Trained MC direction	gN	mN	Trained MC direction
N	16	16	16	16	16	16	16	16	16	16	16	16
Mean direction	310°	280°/100°	348°/168°	19°	281°	191°/11°	14°/104°/ 194°/284°	14°/104°/ 194°/284°	14°/104°/ 194°/284°	69°	339°	227°
Mean vector length	0.227	0.415	0.475	0.168	0.199	0.605	0.327	0.327	0.327	0.350	0.383	0.278
Rayleigh test (P)	0.446	0.062	0.024	0.643	0.540	0.002	0.183	0.183	0.183	0.141	0.095	0.294

See Supplementary Table S2 for explanations.

Supplementary Table S4. Magnetic orientation of zebra finches trained in the presence of a high-intensity, single-frequency 1.4 MHz RF field (RF 1.4_{high}; Set II, test series 3; Fig. 3).

	Birds tested for magnetic compass orientation in the presence of a single-peak 1.4 MHz RF field (RF 1.4_{high}; Fig. 3A)			Birds tested for magnetic compass orientation in the presence of a multi-harmonic 1.4 MHz RF field (RF 1.4_{high+h}; Fig. 3B)			Birds tested for magnetic compass orientation in the ambient RF field (no RF; Fig. 3C)		
Orientation	gN	mN	Trained MC direction	gN	mN	Trained MC direction	gN	mN	Trained MC direction
N	16	16	16	16	16	16	16	16	16
Mean direction	159°/339°	232°/52°	128°	21°/111°/ 201°/291°	21°/111°/ 201°/291°	21°/111°/ 201°/291°	85°/265°	184°	314°/134°
Mean vector length	0.225	0.187	0.485	0.509	0.509	0.509	0.331	0.297	0.411
Rayleigh test (P)	0.451	0.577	0.021	0.014	0.014	0.014	0.174	0.248	0.065

See Supplementary Table S2 for explanations.

Supplementary Table S5. Magnetic orientation of zebra finches trained and tested in the presence of a multi-harmonic, high-intensity 1.4 MHz RF field (RF 1.4_{high+h}; Set II, test series 2b; Fig. 4A), a broadband RF field (RF BB; Set II, test series 2a; Fig. 4B), and in the ambient RF environment (no RF; Set II, test series 4; Fig. 4C).

	Birds trained and tested for magnetic compass orientation in the presence of a multi-harmonic, high-intensity 1.4 MHz RF field (RF 1.4_{high+h}; Fig. 4A)			Birds trained and tested for magnetic compass orientation in the presence of a broadband RF field (RF BB; Fig. 4B)			Birds trained and tested for magnetic compass orientation in the ambient RF field (no RF; Fig. 4C)		
Orientation	gN	mN	Trained MC direction	gN	mN	Trained MC direction	gN	mN	Trained MC direction
N	16	16	16	16	16	16	13	13	13
Mean direction	86°/176°/ 266°/356°	86°/176°/ 266°/356°	86°/176°/ 266°/356°	224°/44°	197°	314°	88°	101°	0°/180°
Mean vector length	0.546	0.546	0.546	0.204	0.314	0.210	0.346	0.389	0.535
Rayleigh test (P)	0.007	0.007	0.007	0.520	0.208	0.502	0.213	0.139	0.021

See Supplementary Table S2 for explanations.
